# Leukocyte mitochondrial DNA copy number and built environment in Mexican Americans: a cross-sectional study

**DOI:** 10.1038/s41598-020-72083-7

**Published:** 2020-09-11

**Authors:** Hua Zhao, Jie Shen, Evan Leung, Xueying Zhang, Wong‐Ho Chow, Kai Zhang

**Affiliations:** 1 Department of Family Medicine and Population Health, School of Medicine, Virginia Commonwealth University, 830 East Main Street, Richmond, VA 23298 USA; 2 Department of Epidemiology, Human Genetics, and Environment Sciences, School of Public Health, The University of Texas, Houston, TX 77030 USA; 3 Department of Epidemiology, The University of Texas MD Anderson Cancer Center, Houston, TX USA

**Keywords:** Biomarkers, Diseases, Risk factors

## Abstract

Mitochondrial DNA (mtDNA) copy number in leukocytes has been regarded as a biomarker for various environmental exposures and chronic diseases. Our previous study showed that certain demographic factors (e.g. age, gender, BMI, etc.) significantly affect levels of leukocyte mtDNA copy number in Mexican Americans. However, the effect of the built environment on leukocyte mtDNA copy number has not been studied previously. In this cross-sectional study, we examined the association between multiple components of the built environment with leukocyte mtDNA copy number among 5,502 Mexican American adults enrolled in Mano-A-Mano, the Mexican American Cohort Study (MACS). Based on the median levels of mtDNA copy number, the study population was stratified into low mtDNA copy number group (< median) and high mtDNA copy number group (≥ median). Among all built environment exposure variables, household density and road/intersection ratio were found to be statistically significant between groups with low and high mtDNA copy number (P < 0.001 and 0.002, respectively). In the multivariate logistic regression analysis, individuals living in areas with elevated levels of household density had 1.24-fold increased odds of having high levels of mtDNA copy number [Odds ratio (OR) = 1.24, 95% confidence interval (CIs) 1.08, 1.36]. Similarly, those living in areas with elevated levels of road/intersection ratio had 1.12-fold increased odds of having high levels of mtDNA copy number (OR = 1.12, 95% CI 1.01, 1.27). In further analysis, when both variables were analyzed together in a multivariate logistic regression model, the significant associations remained. In summary, our results suggest that selected built environment variables (e.g. population density and road/intersection ratio) may influence levels of mtDNA copy number in leukocytes in Mexican Americans.

## Introduction

Molecular epidemiologic studies have shown that mitochondrial DNA (mtDNA) copy number in leukocytes may modify the risk of several types of cancers^1–13^, diabetes^14,15^, cardiovascular disease^16,17^, and aging-related diseases^18,19^, as well as psychological disorders such as stress^20,21^ and depression^22,23^. Thus, mtDNA copy number may serve as an important indicator of health. In our previous analysis using Mano-A-Mano, the Mexican American Cohort study (MACs), we found that higher mtDNA copy number was positively associated with increased risk of all cancer types (adjusted hazard ratio [HR], 1.13; 95% confidence interval [CI] 1.09–1.17). In addition, our study showed that mtDNA copy number in leukocytes was significantly affected by social-demographic characteristics, including age, gender, birth place (Mexico vs US), years of living in the US, body mass index (BMI), and physical activity^13^.

Among Mexican American adults in the MAC study, we reported that living in areas with high density of roads was significantly associated with increased BMI, particularly with BMI ≥ 35^24^. Road density is a factor of the built environment, which includes all of the physical parts of where we live and work (e.g., homes, buildings, streets, open spaces, and infrastructure)^25^. The unique characteristics of the built environment influence a person’s level of physical activity. For example, inaccessible or nonexistent sidewalks, bicycle paths, and walking paths contribute to sedentary habits^26^, which may consequently lead to poor health outcomes, including obesity, cardiovascular disease, diabetes, and some types of cancer. The influence of built environment on health can also significantly vary among a population^25^. Compared to their White counterparts, Mexican Americans experience health outcomes that are more vulnerable to changes in the built environment due to their lower socioeconomic status^27^. Given the observed significant associations of road density with body mass index (BMI)^24^ and mtDNA copy number with BMI and physical activity^13^, we were driven to assess whether the built environment may modify mtDNA copy number in leukocytes. In our previous analysis, we found that birth place and years of living in the US were associated with mtDNA copy number. The built environment is also affected by socioeconomic status, which is closely linked to immigration and acculturation. Therefore, the relationships among the built environment and mtDNA copy number may be further modified by immigration and acculturation.

The objective of this study is to estimate the cross-sectional associations between selected components of the built environment and mtDNA copy number in leukocytes among adult participants enrolled in the MAC study, a large ongoing Mexican American cohort study in the US^28^. Built environment factors include physical activity environment, land use, and food environment^24^. We hypothesize that built environment factors may modify leukocyte mtDNA copy number.

## Methods

### Study population

The samples used in this study were obtained from individuals enrolled in the MAC study, a large population-based cohort of Mexican-origin households recruited in the Houston, Texas area. The MAC study was initiated in July 2001 for an ongoing prospective study of first- and second-generation Mexican-origin immigrant households in Houston. It is maintained by the Department of Epidemiology at The University of Texas MD Anderson Cancer Center. A detailed description of the sampling and recruitment strategy has been published previously^28^. In this study, we included 5,502 participants from the MAC study. The inclusion criteria included: age greater than 20 years old; no reported cancer, diabetes, or cardiovascular diseases at the time of cohort entry; and collected data on leukocyte mtDNA copy number. The study was approved by the Institutional Review Board of MD Anderson Cancer Center. All methods were performed in accordance with the relevant guidelines and regulations.

### Determination of mtDNA copy number

We used a real-time quantitative polymerase chain reaction (PCR) to determine mtDNA copy number. The detailed method and data are detailed in our previous publication^13^.

### Built environment factors

The data on built environment factors are presented in our previous publication^24^. In brief, the factors represented three important domains of the built environment: physical activity environment, land use variety, and food environment. In physical activity environment, we included five exposure variables [population density (people per km^2^), household density (households per km^2^), road density (number of road links per km^2^), intersections density (number of road intersections per km^2^), and the ratio of roads versus intersections] within a half-mile radius surrounding each participant's residence. Additionally, we considered the nearest parks and the highways around each participant's residential address. For land use, we used the index of land use mix based on the published method by Rundle et al.^29^. Food environment was measured as the weighted Modified Retail Food Environment Index (mRFEI) published by the Centers for Diseases Control and Prevention (CDC).

### Statistical analysis

Log transformed mtDNA copy number was used in the analysis. Levels of demographic and built environment variables were grouped into two categories based on the median levels of mtDNA copy number: low mtDNA copy number and high mtDNA copy number. Descriptive statistics (e.g. median and range) were calculated for demographic and built environmental variables in each category and were further compared between the two categories. The Wilcoxon Rank Sum test was applied for the comparison. False discovery rate (FDR) test was applied to assess the multiple comparisons. For each built environment variable, we further applied multivariate logistic regression analysis to assess its relationship with mtDNA copy number. Backward stepwise selection was applied to select the covariates. The final regression model included age (continuous), sex (men vs women), BMI (continuous), physical activity (high, median, vs low), health insurance (yes vs no), and the interaction term of age versus sex as covariates. We examined physical activity using (1) survey instruments from the 2007 to 2008 National Health and Nutrition Examination Survey (NHANES; CDCP, 2007) that assess the frequency and duration of respondents’ moderate and vigorous recreational, occupational, and transport-related physical activity and (2) supplemental items following the NHANES format that inquire about activities performed in and around the home. For each tested built environment variable, the variable was first classified into four quartiles, based on 25%, 50% and 75% levels of each variable, and then dichotomized into two categories: low (1st quartile) and high (quartiles 2–4 combined). We estimated odds ratios (ORs) and 95% confidence intervals (CIs) for associations between each tested built environmental variable and the mtDNA copy number. Finally, we included built environment variables significantly associated with mtDNA copy number as well as demographic variables together in a multivariate logistic regression model. A similar approach was applied to select covariates.

### Ethics approval and consent to participate

The study was approved by the Institutional Review Board of MD Anderson Cancer Center. All methods were performed in accordance with the relevant guidelines and regulations. Written consent has been obtained from all participants.

## Results

A total of 5,502 study participants were included in this study. We divided the study population into two groups based on the median levels of mtDNA copy number: low mtDNA copy number (N = 2,751) and high mtDNA copy number (N = 2,751). Basic demographics of each group are shown in Table 1. Compared to those with low mtDNA copy number, those with high mtDNA copy number were younger (median: 37 vs 38, P = 0.002), more likely to be women (P < 0.001), and less likely to have high levels of acculturation (P = 0.016). In addition, BMI was statistically significantly lower in those with high mtDNA copy number than in those with low mtDNA copy number (median: 30.07 vs 30.92, P < 0.001). The current study incorporated ten built environment variables, including population density, household density, intersection density, road density, road/intersection ratio, distance to highway, walking time to the nearest park, networked distance to the nearest park, Rundle’s LUM, and CDC mRFEI. The respective median levels and range in low and high mtDNA copy number categories are listed in Table 1. We found that median levels of household density were statistically significantly higher in those with high mtDNA copy number compared to their counterparts (median: 0.80 vs 0.78, P < 0.001). Similarly, median levels of road/intersection ratio were statistically significantly higher in those with high mtDNA copy number compared to their counterparts (median: 0.56 vs 0.52, P = 0.002). The association remained significant after FDR adjustment (q value = 0.002 and 0.011, respectively). No significant association was observed for other built environment variables.

**Table 1:** Descriptive statistics of built environment variables, mtDNA copy number, and covariates of 5,502 Mexican American adults enrolled in the MAC study.

Variables	Low mtDNA copy number group		High mtDNA copy number group		P value
	Median	Range	Median	Range	
Age	38	16, 85	37	15, 81	0.002
BMI (kg/m^2^)	30.92	9.80, 74.52	30.07	16.97, 70.99	< 0.001
Population density (1,000 people per km^2^)	2.33	0.15, 6.11	2.37	0.08, 5.80	0.433
Household density (1,000 households per km^2^)	0.78	0.02, 2.90	0.80	0.05, 3.30	< 0.001
Intersection density (100 intersections per km^2^)	3.08	0.40, 8.52	3.05	0.14, 7.92	0.26
Road density (100 roads per km^2^)	5.67	0.61, 16.15	5.61	0.25, 15.01	0.229
Road/intersection ratio (number per km^2^)	0.52	0.51, 0.64	0.56	0.53, 0.66	0.002
Distance to highway (km)	0.92	4.06E − 04, 7.23	0.95	4.06E − 04, 10.78	0.766
Walking time to the nearest park (min)	9	1, 99	9	1, 116	0.431
Networked distance to the nearest park (km)	0.70	1.00E − 03, 8.20	0.70	1.00E − 03, 9.80	0.477
Rundle’s LUM (0–1)	0.33	1.00E − 03, 0.99	0.33	1.00E − 03, 1.00	0.876
CDC mRFEI (0–100)	7.85	0, 37.45	8.02	0, 37.42	0.131

	Number	Percentage (%)	Number	Percentage (%)	
**Sex**					
Men	630	22.90	471	17.12	
Women	2,121	77.10	2,280	82.88	< 0.001
**Place of birth**					
Mexico	2,120	77.12	2,176	79.13	
US	629	22,88	574	20.87	0.167
**Insurance**					
No	1,416	51.47	1,438	52.29	
Yes	1,335	48.53	1,312	47.71	0.543
**Physical activity**					
Low	1,888	69.49	1,916	70.57	
Median	708	26.06	710	26.15	
High	121	4.45	89	3.28	0.079
**Acculturation**					
Low	1,694	62.03	1,784	65.16	
High	1,037	37.97	954	34.84	0.016

To further assess the relationships between built environment variables and mtDNA copy number, we applied multivariate logistic regression analysis. We treated built environment variables as categorical variables, first by quartile and then dichotomized (high vs low: quartile 4 vs quartiles 1–3) (Table 2). In quartile analysis, compared to those at 1st quartile (the lowest level) of household density, those at 3rd and 4th quartiles had 1.20- and 1.31-fold increased odds of having high levels of mtDNA copy number (OR = 1.20, 95% CI 1.03, 1.40; OR = 1.31, 95% CI 1.11, 1.48, respectively). The covariates included age, sex, age × sex, BMI, physical activity, and health insurance. A dose–response relationship was also observed (P for trend = 0.016). A similar association was observed for road/intersection ratio. Compared to those at 1st quartile, those at 3rd and 4th quartiles had 1.14- and 1.17-fold increased odds of having high levels of mtDNA copy number (OR = 1.14, 95% CI 1.00, 1.32; OR = 1.17, 95% CI 1.01, 1.37, respectively). A dose–response relationship was also observed (P for trend = 0.030). In the dichotomized analysis, we combined quartiles 2, 3, and 4 together. Compared to those at 1st quartile, those with higher levels of household density had 1.24 fold increased odds of having high levels of mtDNA copy number (OR = 1.24, 95% CI 1.08, 1.36), and those with higher levels of road/intersection ratio had 1.12 fold increased odds of having high levels of mtDNA copy number (OR = 1.12, 95% CI 1.01, 1.27). No significant association was observed for other built environment variables.

**Table 2:** Logistic regression analysis to estimate the association between built environment variable and mtDNA copy number.

Variables	ORs (95% CI)^a^	ORs (95% CI)^a^
**P value Population density**		
1st quartile	1.00	1.00
2nd quartile	1.11 (0.95, 1.29)	
3rd quartile	1.15 (0.99, 1.35)	1.11 (0.98, 1.25)
4th quartile	1.06 (0.91, 1.23)	
	P for trend = 0.418	
**Household density**		
1st quartile	1.00	1.00
2nd quartile	1.03 (0.89, 1.20)	
3rd quartile	1.20 (1.03, 1.40)	1.24 (1.08, 1.36)
4th quartile	1.31 (1.11, 1.48)	
	P for trend = 0.016	
**Intersection density**		
1st quartile	1.00	1.00
2nd quartile	1.06 (0.91, 1.23)	
3rd quartile	1.03 (0.89, 1.20)	1.00 (0.88, 1.13)
4th quartile	0.91 (0.78, 1.06)	
	P for trend = 0.239	
**Road density**		
1st quartile	1.00	1.00
2nd quartile	1.07 (0.92, 1.25)	
3rd quartile	1.05 (0.90, 1.22)	1.01 (0.89, 1.15)
4th quartile	0.92 (0.79, 1.07)	
	P for trend = 0.289	
**Road/intersection ratio**		
1st quartile	1.00	1.00
2nd quartile	1.07 (0.92, 1.24)	
3rd quartile	1.14 (0.97, 1.32)	1.12 (1.01, 1.27)
4th quartile	1.17 (1.01, 1.37)	
	P for trend = 0.030	
**Distance to highway**		
1st quartile	1.00	1.00
2nd quartile	1.03 (0.89, 1.20)	
3rd quartile	1.14 (0.98, 1.32)	1.05 (0.93, 1.19)
4th quartile	0.99 (0.85, 1.16)	
	P for trend = 0.756	
**Walking time to the nearest park**		
1st quartile	1.00	1.00
2nd quartile	1.00 (0.86, 1.17)	
3rd quartile	1.03 (0.88, 1.21)	0.98 (0.86, 1.12)
4th quartile	0.91 (0.78, 1.06)	
	P for trend = 0.282	
**Networked distance to the nearest park**		
1st quartile	1.00	1.00
2nd quartile	0.99 (0.85, 1.17)	
3rd quartile	1.07 (0.91, 1.25)	0.99 (0.87, 1.13)
4th quartile	0.92 (0.79, 1.08)	
	P for trend = 0.400	
**Rundle's LUM**		
1st quartile	1.00	1.00
2nd quartile	0.87 (0.75, 1.01)	
3rd quartile	1.01 (0.87, 1.17)	0.93 (0.82, 1.05)
4th quartile	0.92 (0.79, 1.07)	
	P for trend = 0.659	
**CDC mRFEI**		
1st quartile	1.00	1.00
2nd quartile	1.07 (0.92, 1.25)	
3rd quartile	1.09 (0.93, 1.26)	1.10 (0.97, 1.25)
4th quartile	1.14 (0.98, 1.33)	
	P for trend = 0.097	

^a^Adjusted by age, sex, age × sex, BMI, physical activity, and health insurance.

Next, we incorporated both household density and road/intersection ratio in a logistic regression model to assess their relationships with mtDNA copy number (Table 3). The model also included age, sex, age × sex, BMI, physical activity, and health insurance. Both household density and road/intersection ratio were treated as quartile categorical variables. We found that increased household density was associated with high mtDNA copy number. Compared to those at 1st quartile (the lowest level) of household density, those at 3rd and 4th quartiles had 1.23- and 1.31-fold increased odds of having high levels of mtDNA copy number (OR = 1.23, 95% CI 1.05, 1.44; OR = 1.31, 95% CI 1.10, 1.54, respectively). A dose–response relationship was also observed (P for trend = 0.012). When treated as the binary variables, similarly, high household density was associated with 1.28-fold increased odds of having high levels of mtDNA copy number (OR = 1.28, 95% CI 1.12, 1.48). A significant association was also observed for road/intersection ratio, but only for the 4th quartile. Compared to those at 1st quartile (the lowest level) of road/intersection ratio, those at 4th quartile had 1.21-fold increased odds of having high levels of mtDNA copy number (OR = 1.21, 95% CI 1.03, 1.42). A significant trend was also observed (P for trend = 0.025). When treated as the binary variables, similarly, high household density was associated with 1.13-fold increased odds of having high levels of mtDNA copy number (OR = 1.13, 95% CI 1.00, 1.28).

**Table 3:** Logistic regression analysis to estimate the association between built environment variable and mtDNA copy number.

Variables	ORs (95% CI)^a^	P value
Age	0.96 (0.94, 0.98)	< 0.001
Gender (female vs male)	0.61 (0.40, 0.96)	0.032
Age × gender	1.02 (1.01, 1.03)	< 0.001
BMI	0.98 (0.97, 0.99)	< 0.001
Physical activity		
Medium vs low	0.98 (0.86, 1.14)	0.775
High vs low	0.74 (0.55, 0.99)	0.040
Insurance	0.90 (0.80, 0.99)	0.048
Household density		
1st quartile	1	
2nd quartile	1.09 (0.93, 1.28)	0.277
3rd quartile	1.23 (1.05, 1.44)	0.009
4th quartile	1.31 (1.10, 1.54)	0.007
	P for trend = **0.012**	
Road/intersection ratio		
1st quartile	1	
2nd quartile	1.07 (0.92, 1.25)	0.375
3rd quartile	1.12 (0.96, 1.31)	0.143
4th quartile	1.21 (1.03, 1.42)	0.018
	P for trend = 0.025	

^a^Adjusted by age, sex, age × sex, BMI, physical activity, and insurance.

## Discussion

In contrast to nuclear DNA, mtDNA is vulnerable to reactive oxygen species (ROS) damage due to the lack of histone protection or effective DNA repair mechanisms^30,31^. Damage to mtDNA alters the copy number level of mtDNA and results in mitochondrial dysfunction, which is considered to be an important pathogenesis of various types of chronic diseases^32^. The mtDNA copy number may therefore reflect the level of mtDNA damage and consequently mitochondrial function^17^. Thus, mtNDA copy number is an intermediate biomarker which can not only reflect environmental exposure, but may also contribute to chronic diseases. In our previous study, we found that social-demographic variables (e.g. age, sex, BMI, physical activity, birth place, and duration of living in US) could modify levels of leukocyte mtDNA copy number in Mexican Americans^13^. However, to date, no study has ever analyzed the influence of built environment variables on leukocyte mtDNA copy number.

In this study, we found that median levels of household density and road/intersection ratio were higher in those with high mtDNA copy number compared to those with low mtDNA copy number (P < 0.001 and 0.002, respectively). In logistic regression analysis, we found that increased household density and road/intersection ratio were associated with increased odds of having high levels of mtDNA copy number in Mexican Americans. The results from this study indicate that built environment variables influence leukocyte mtDNA copy number. Future study is needed to further assess whether leukocyte mtDNA copy number may mediate the association between built environment and chronic diseases.

Higher household density may correlate with more urban living and subsequently indicate more walkable communities with higher levels of physical activity. As higher levels of physical activity are known to positively correlate with mtDNA copy numbers, increased household density may lead to higher mtDNA copy numbers. This hypothesis may explain the observed positive association between household density and mtDNA copy number. Interestingly, we did not observe significant correlations between household density with physical activity and BMI, which were associated with mtDNA copy number, suggesting that the effect of household density on mtDNA copy number is independent of physical activity and BMI.

High road/intersection ratio usually indicates less road connectivity, which implicates a less-walkable built environment. However, in our analysis, we adjusted for BMI and physical activity. Therefore, the positive association between road/intersection ratio and mtDNA copy number is unlikely due to the effect of physical activity. High road/intersection ratio values may also reflect less stop-and-go traffic and less congestion, which lead to less traffic-related air pollution. In a recent study, Breton et al. reported that traffic-related air pollution can directly affect mitochondrial respiratory function and decrease mtDNA copy number in blood DNA^33^. In another study in China, decreased blood mtDNA copy number was associated with increased exposure to black carbon during work hours and short-term exposure to PM_10_^34^. Furthermore, a significant variation in traffic-related air pollution has been observed in the area in where our study participants reside^35^. Thus, findings from previous literature support the reverse association between traffic-related air pollution and mtDNA copy number.

Interestingly, the positive associations of household density and road/intersection ratio with mtDNA copy number was only observed among non-obese study participants. BMI is one of the most significant factors associated with mtDNA copy number^13^. In our previous study, we found that several built environment factors, including road density, intersection density, networked distance, and walking time to the nearest parks, had statistically significantly linear associations with BMI^24^. It is possible that the strong associations of BMI with built environmental factors and mtDNA may overshadow the modest association between road/intersection ratio and mtDNA copy number. We also found that the associations were only observed among those born in Mexico. Given that only about 21% of study participants were born in the US, the lack of association among those born in the US may be due to the small sample size. Additionally, we found that the positive association between road/intersection ratio and mtDNA copy number was only observed among those living in the US for at least 10 years. This finding may be due to the immigrant heath effect^36^, where newly- or recently-immigrated individuals are more likely to maintain their health advantage and accumulate adverse effects from environment exposure more slowly. Furthermore, newly- or recently-immigrated individuals tend to live in more isolated neighborhoods. Thus, we may not observe enough variations in terms of built environment factors among those living in the US for less than 10 years.

Our study is subject to several limitations. First, we examined the cross-sectional links between exposure to the built environment and mtDNA copy number. One common drawback of using a cross-sectional design is that we cannot infer the causal relationship between the exposure and outcome. Second, the demographic data used in our study were self-reported. Third, mtDNA copy number in leukocytes may modify the risk of several types of chronic diseases and conditions^1–23^ as well as biobehavioral factors, but the direction and strength of each association differs significantly. Thus, it is difficult to determine the exact underlying cause of each observed association and its potential health outcomes. Despite these limitations, our study is the first to assess the potential biological effect of the built environment on intermediate biomarkers.

## Conclusions

We found that those living in areas with high levels of household density and road/intersection ratio are more likely to have high levels of leukocyte mtDNA copy number. The results from this study need to be further validated in future studies.

## Data Availability

The datasets used and/or analyzed during the current study are available from the corresponding author on reasonable request.

## Abbreviations

mtDNA: Mitochondrial DNA
MACS: The Mexican American Cohort Study
BMI: Body mass index
OR: Odds ratio
CI: Confidence interval
